# Dr. Charles Christian Dellenbaugh

**Published:** 1917-02

**Authors:** 


**Dr. Charles Christian Dellenbaugh**, Buffalo 1859, died at his home in Portland, Mich., Dec. 25, 1916, aged 82. He had practiced in Portland for over 50 years. By error, he was starred as dead in the Alumni Catalogue of the Medical Dept. of the University of Buffalo, when it was issued in Oct. 1915. We request information of alumni whose address is unknown and of whom it is not even known whether they are dead or alive. In fact, we are always glad to receive information correcting errors or supplying omissions in either this catalogue or the State Directory.

